# Acute isometric and dynamic exercise do not alter cerebral sympathetic nerve activity in healthy humans

**DOI:** 10.1177/0271678X241248228

**Published:** 2024-04-13

**Authors:** Michael M Tymko, Audrey Drapeau, Maria Augusta Vieira-Coelho, Lawrence Labrecque, Sarah Imhoff, Geoff B Coombs, Stephan Langevin, Marc Fortin, Nathalie Châteauvert, Philip N Ainslie, Patrice Brassard

**Affiliations:** 1 Integrative Cerebrovascular and Environmental Physiology SB Laboratory, Department of Human Health and Nutritional Sciences, College of Biological Science, University of Guelph, Guelph, Canada; 2 Department of Medicine, Faculty of Medicine, University of British Columbia, Vancouver, Canada; 3 Department of Kinesiology, Faculty of Medicine, Université Laval, Québec, Canada; 4 Institut universitaire de cardiologie et de pneumologie de Québec-Université Laval, Québec, Canada; 5 Department of Biomedicine, Pharmacology and Therapeutics Unit, Faculty of Medicine, University of Porto, Portugal; 6 Department of Psychiatry and Mental Health, University Hospital Center of São João, Porto, Portugal; 7 Department of Kinesiology, Faculty of Science, McMaster University, Hamilton, Canada; 8 Centre for Heart, Lung and Vascular Health, School of Health and Exercise Sciences, University of British Columbia – Okanagan, Kelowna, Canada

**Keywords:** Cerebral blood flow, sympathetic nervous activity, noradrenaline spillover

## Abstract

The impact of physiological stressors on cerebral sympathetic nervous activity (SNA) remains controversial. We hypothesized that cerebral noradrenaline (NA) spillover, an index of cerebral SNA, would not change during both submaximal isometric handgrip (HG) exercise followed by a post-exercise circulatory occlusion (PECO), and supine dynamic cycling exercise. Twelve healthy participants (5 females) underwent simultaneous blood sampling from the right radial artery and right internal jugular vein. Right internal jugular vein blood flow was measured using Duplex ultrasound, and tritiated NA was infused through the participants' right superficial forearm vein. Heart rate was recorded via electrocardiogram and blood pressure was monitored using the right radial artery. Total NA spillover increased during HG (P = 0.049), PECO (P = 0.006), and moderate cycling exercise (P = 0.03) compared to rest. Cerebral NA spillover remained unchanged during isometric HG exercise (P = 0.36), PECO after the isometric HG exercise (P = 0.45), and during moderate cycling exercise (P = 0.94) compared to rest. These results indicate that transient increases in blood pressure during acute exercise involving both small and large muscle mass do not engage cerebral SNA in healthy humans. Our findings suggest that cerebral SNA may be non-obligatory for exercise-related cerebrovascular adjustments.

## Introduction

Several physiological factors, such as aerobic/resistance exercise and rapid eye movement sleep, as well as pathological clinical conditions including autonomic dysreflexia, baroreflex failure, or uncontrolled arterial hypertension, require efficient counter-regulation against transient arterial blood pressure fluctuations. Without the neuroprotective influences of sympathetic activation or cerebral autoregulation (which refers to the relationship between cerebral perfusion pressure and blood flow during changes in arterial blood pressure), acute surges in arterial blood pressure could increase the risk of hyper-perfusion injury, predisposing to stroke or blood-brain barrier breakdown.^1^ As such, tight regulation of cerebral blood flow (CBF) during acute changes in arterial blood pressure helps to maintain normal cerebral function.^2–4^

Cerebral sympathetic nerve activity (SNA) increases with acute increases in arterial blood pressure induced pharmacologically or mechanically in anesthetized lambs, but does not change with acute reductions in arterial blood pressure.^5^ This finding suggests that elevations in cerebral SNA could serve as the effective dynamic regulator of CBF during transient increases in arterial blood pressure, at least in this animal model. However, much less work has been conducted in clinical and healthy human models. In humans with essential arterial hypertension, cerebral noradrenaline (NA) release is elevated,^6^ which in support of the existing animal literature, could be interpreted as a cerebral protective mechanism for overperfusion, or alternatively, elevated cerebral NA release itself could contribute to chronic arterial hypertension. A study in healthy humans demonstrated that moderate intensity cycling exercise, which is known to acutely increase arterial blood pressure, CBF, and peripheral SNA,^7^ resulted in no change in cerebral NA spillover, an index of cerebral specific SNA.^8,9^ The latter finding in healthy humans indicates that cerebral SNA does not contribute to CBF regulation during moderate intensity aerobic exercise; however, these data have yet to be replicated, and the effects of isometric exercise [e.g., handgrip (HG)] and post-exercise circulatory occlusion (PECO), which increases sympathetic outflow independent from exercise, has not been explored. A better understanding of how acute physiological stresses that are known to result in increased peripheral SNA and arterial blood pressure, such as dynamic cycling exercise, HG exercise, and PECO, may provide the basis for developing new approaches to the management of pathological conditions that require attenuation of CBF in arterial hypertension and/or loss of cerebrovascular regulation in humans. Each of these modalities to increase peripheral SNA and arterial blood pressure are distinct from one another (e.g., dynamic vs isometric exercise, large vs small muscle activation, active vs inactive tissue), potentially giving insight into whether different types of SNA activation can influence cerebral SNA.

The purpose of the current study was to measure the effects of an acute increase in arterial blood pressure using isometric HG exercise and PECO and dynamic cycling exercise on cerebral SNA using the NA spillover technique. Based on previous work in humans,^7^ we hypothesized that cerebral NA spillover would not change during both isometric and dynamic exercise, highlighting that cerebral SNA is not a regulatory mechanism during moderate intensity exercise in healthy humans.

## Methods

### Ethical approval and informed consent

The present study was approved by the *Comité d’éthique de la recherche de l’Institut universitaire de cardiologie et de pneumologie de Québec* (CER: 21557). All participants provided their written informed consent prior to participating. The study conformed to the standards set by the Tri-Council Policy Statement: Ethical Conduct for Research Involving Humans (TCPS 2) and the Declaration of Helsinki, except for registration in a database. The data that support the findings of this study are available from the corresponding author upon reasonable request.

### Participants

Twelve young and healthy participants (five females) were recruited to participate in this study. A sample size calculation revealed that a minimum of 5 participants would be necessary to yield a statistically significant increase in arterial blood pressure between baseline and steady-state isometric HG exercise with a power of 0.80, a p value <0.05 and a Cohen’s d effect size of 1.86.^10^ Twelve participants were recruited to consider potential participant drop out. Pilot testing revealed that a similar increase (∼20%) in mean arterial blood pressure compared to isometric HG exercise was achievable during our dynamic supine cycling exercise protocol. All participants were recruited at the Université Laval campus (Québec City, Canada). Participants were free of known cardiovascular, respiratory and neurological disease, and non-smokers. Participants were not on any current medication except for oral contraceptives (n = 2). For unforeseen methodological issues (e.g., catheter obstruction [n = 1], and equipment failure [n = 2]), some participants did not complete the cycling portion of the study.

### Experimental design

On an initial visit, participants came to the laboratory to have all procedures explained to them in person, sign informed consent to the study, complete three left-handed maximal HG exercise (for the evaluation of their maximal voluntary contraction), and have their internal jugular veins (IJV) scanned via Duplex ultrasound to ensure adequate image quality for this vessel. On the second experimental visit, participants arrived at the laboratory having abstained from alcohol, caffeine and exercise for ≥12 hrs, and were fasted for a minimum of 2 hrs, but allowed to drink water ad libitum. All testing procedures were completed in ∼5 hrs. Seven participants were tested in the morning (7 am), and 5 participants were tested in the afternoon (12 pm). Data collection took place over a period of two weeks.

**Figure 1.**
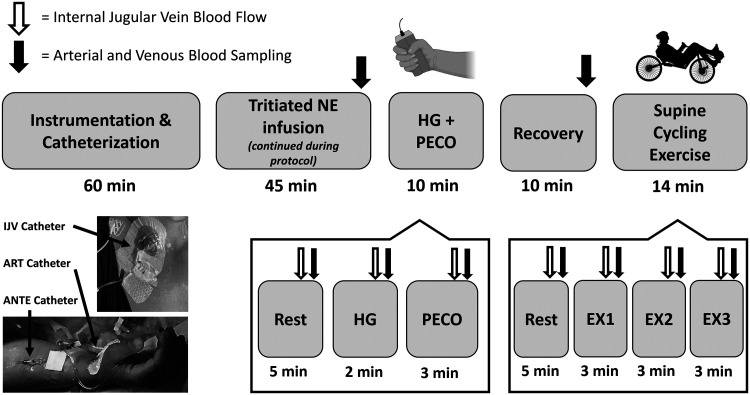
Schematic of the experimental protocol. Participants were instrumented with IJV, arterial (i.e., ART), and superficial vein of the forearm (i.e., ANTE) catheters upon arrival to the laboratory. The experimental protocol began with a 45-min infusion of tritiated NA (to steady-state) that continued throughout the entire protocol. The participants were then asked to perform a 2-min isometric handgrip test at 30% of their maximal voluntary contraction, which was followed by a 3-min post-exercise circulatory occlusion test. After a 10-min recovery, participants then performed a submaximal supine cycling exercise test that consisted of three stages for both male (75 W, 100 W, 125 W) and female (50 W, 75 W, 100 W) participants.

Under sterilized conditions, participants had their right radial artery, right superficial forearm vein, and right IJV catheterized (see additional details below). After catheterization, the participants were transferred to the laboratory, placed supine, and rested for 1 hr to offset the nociceptive stimuli associated with catheterization. Participants were then instrumented for data collection (see details below). A continuous tracer infusion of ^3^H-labelled L-NA with high specific activity (10 Ci mmol^−1^), in order to avoid an elevation in arterial blood pressure and reflex lowering NA release,^11^ was administered intravenously at 0.8 µCi min^−1^ via a superficial vein of the right forearm to steady-state (45 min). The exercise data presented in this study were part of a larger series of separate experiments including lower body negative pressure without and with hypercapnia. After a 5-min resting period, the participants first completed a two-min isometric HG test to 30% of their maximal voluntary contraction of their left hand (previously measured). Participants completed the HG test in a supine position with a hand dynamometer connected to an acquisition system (Biopac System, Santa Barbara, California, USA) while seeing a screen on which the individual target workload (i.e. 30% of their maximal voluntary contraction) was marked by a horizontal red line. To ensure this target workload was maintained over the 2 min, an experimenter was placed closed to the screen and provided constant encouragement for participants to stay as close as possible to the target workload. Participants were instructed to avoid doing a Valsalva maneuver during the HG protocol. Immediately after the isometric HG test, a PECO test was completed by inflating a pressure cuff around the forearm to ∼250 mmHg for three min. The PECO was conducted in order to assess the influence of increased global SNA via local metabolic afferent activity (i.e., muscle metaboreflex).^12^ After the HG and PECO tests, the participants were then instructed to rest quietly for 10 min to recover. The participants feet were then placed into the pedals of a supine cycle ergometer attached to the hospital bed (LODE Lode BV, The Netherlands). After a 5-min resting period, the participants cycled at three exercise workloads that represented mild-to-moderate exercise for young and healthy males and females (75, 100, and 125 Watts for males; 50, 75, and 100 Watts for females), with each intensity lasting three min (Figure 1). Higher intensities of exercise were avoided due to concerns of obtaining reliable ultrasound measures. These exercise workloads are referred to as EX1, EX2, and EX3.

### Experimental measurements

#### Cardiovascular measurements

All continuously recorded cardiovascular measurements were acquired at 1000 Hz using an analog-to-digital converter (Powerlab/16SP ML 880; ADInstruments, Colorado Springs, CO, USA) interfaced with a personal computer. Commercially available software was used to analyse cardiovascular variables (LabChart V7.1; ADInstruments). Electrocardiogram electrodes were placed in lead II configuration (Bioamp, ML132; ADInstruments) to measure heart rate. The radial artery catheter was connected to a commercially available arterial blood sampling kit integrated with a pressure transducer (VAMP Adult, Edwards Lifescience, Irvine, CA), allowing for beat-by-beat measurements of arterial blood pressure. The radial arterial blood pressure waveform was corrected to brachial artery blood pressure using a manual sphygmomanometer. A one-min average was taken for these variables at the end of the rest period, during the isometric HG protocol (HG and PECO), and each of the cycling exercise stages (EX1, EX2, EX3).

#### Respiratory measurements and dynamic end-tidal forcing

Participants breathed through a mouthpiece while wearing a nose clip. Both partial pressure of end-tidal carbon dioxide (P_ET_CO_2_) and oxygen (P_ET_O_2_) were sampled at the mouth and recorded by a calibrated gas analyzer (model ML206, ADInstruments); breathing frequency, tidal volume, and minute ventilation were measured by a pneumotachograph (model HR 800 L, Hans Rudolph) connected in series to a bacteriological filter. P_ET_CO_2_ and P_ET_O_2_ were controlled by a portable dynamic end-tidal forcing system during the HG and PECO protocol. This system was not used to control end-tidal gases during cycling exercise, since the available system could not accommodate the high respiratory flow rates associated with cycling exercise. Details on this device are described in detail elsewhere.^13,14^ This system has been validated (against arterial blood gases) and used to control end-tidal gases during several different physiological stressors. A one-min average was taken for these variables at the end of the rest period, during the isometric HG protocol (HG and PECO), and each of the cycling exercise stages (EX1, EX2, EX3).

#### Extracranial blood flow

Simultaneous B-mode and Doppler ultrasonography were performed to assess continuous diameter and blood velocity recordings of the right IJV using a 10 MHz linear array probe attached to a high-resolution ultrasound machine (Terason t3200; Teratech, Burlington, MA, USA). The same experienced sonographer insonated the right IJV for all participants. The right IJV was preferred since blood flow is usually greater in the right IJV compared to the left IJV. Of importance, this asymmetry in IJV drainage has no effect on brain NA spillover.^15^ The insonation angle remained unchanged at 60 degrees throughout the entire protocol. Ultrasound recordings were screen captured and saved for offline analysis. Blood flow analyses was performed using edge-detection software, which allows for the integration of synchronous diameter and velocity measurements to determine the mean beat-to-beat flow at 30 Hz independent of investigator bias.^16^ Mean blood flow was determined as half of the time-averaged maximal velocity multiplied by the cross-sectional luminal area for a minimum of 12 cardiac cycles.^17^ A one-min average, acquired from the final minute of each stage, was taken for these variables at the end of the rest periods, during the isometric HG protocol (HG and PECO), and each of the cycling exercise stages (EX1, EX2, EX3). IJV conductance was calculated using the following equation:

(1) IJV conductance=IJV  blood  flow/mean  arterial  pressure

#### Arterial and venous catheterization

Three catheters were placed under sterile conditions prior to the experimental protocol: 1) an 18-gauge venous catheter into a superficial vein of the right forearm; 2) a 20-gauge arterial catheter (Arrow, Markham, ON, Canada) into the right radial artery, and 3) a 16-gauge central venous catheter (Arrow, Markham, ON, Canada) into the right IJV, which was advanced cephalad to the jugular bulb. Placement of the IJV was confirmed with an IJV oxygen saturation (S_J_O_2_) of <75%.

#### Arterial and venous blood analysis

Before sampling was started, the dead space volume was withdrawn and then an arterial sample (3 ml) was collected in pre-heparinized syringes (safePICO syringes, Radiometer, Copenhagen, Denmark). Blood samples were taken at the end of the rest periods, during the HG protocol (HG and PECO), and each of the cycling exercise stages (EX1, EX2, EX3). Air bubbles were immediately evacuated from the syringe, and blood gas analysis was performed within 5 min of sampling with a gas analyzer (ABL80 FLEX, Radiometer). The blood gas analyzer underwent a one-point calibration every 4 hrs, 2-point calibration every 8 hrs, and an additional automated 1-point calibration every 15 samples using manufacturer's standard quality checks, and ampule-based quality checks. All blood data were analyzed assuming a core body temperature of 37.0°C. Reported variables that were calibrated and analyzed included hemoglobin concentration ([Hb]), (arterial) hematocrit (Hct), oxygen saturation (SO_2_), partial pressure of oxygen (PO_2_), partial pressure of carbon dioxide (PCO_2_), pH, glucose, and lactate. Furthermore, arterial (CaO_2_) and venous (CvO_2_) oxygen content were calculated using the following equation:

(2) $$\text{Oxygen}\;\text{content}\left(\text{mL}/\text{dL}\right)=\left(1.34\times \left[\text{Hb}\right]\times \left({\text{SO}}_{2}/1\mathrm{00}\right)\right)+\left(0.00\mathrm{31}\times {\text{PO}}_{2}\right)$$

#### Neurochemical assays

The assay of NA in plasma samples by means of high‐performance liquid chromatography with electrochemical detection (HPLC-EC) was performed adapted from previously described methods.^8,18^ In brief, aliquots of 1000 µl of plasma were placed in 5‐ml conical‐based glass vials with 50 mg alumina, and the pH of the samples was adjusted to 8.6 by the addition of Tris buffer. Dihydroxybenzylamine hydrobromide was used as internal standard. The absorbed catecholamines were then eluted from the alumina with 300 µl of 0.2 M perchloric acid on Costar Spin‐X microfilter tubes. From this eluate, 50 µl was injected into a high‐pressure liquid chromatograph (Gilson Medical Electronics, Villiers le Bel, France) and 150 µl were used for [^3^H]NE radioactivity measure.

#### [^3^H]NA-radioactivity measure

Radioactivity was measured by liquid scintillation counting (liquid scintillation counter 1209 Rackbeta, LKB Wallac, Turku, Finland) in 150 µl aliquots from alumina eluate after addition of 10 ml of scintillation mixture (OptiPhase “HiSafe” 3, LKB, Loughborough, England). Final concentrations were corrected for loss during extraction using recoveries of internal standards.

##### Noradrenaline Spillover

At steady-state during the infusion of tritiated NA, the rate of spillover of NA into plasma and total NA clearance were calculated using the following equations:^11^

(3) NA clearance=[ 3H]NA  infusion  rate (dpm/min)/plasma [ 3H] NA concentration (dpm/ml)

(4) NA spillover=[ 3H]NA  infusion  rate (dpm/min)/plasma  NA  specific  activity (dpm/pg)

The rate of cerebral NA spillover was calculated using the Fick principle, which was corrected for the fractional extraction of [^3^H]NA:^19^

(5) Cerebral  NA  spillover=[(IJV [NA]–Arterial [NA])+(Arterial [NA]×fractional  extraction  of  cerebral [3H] NA)]×IJVplasma  flow

where IJV plasma flow represents IJV blood flow multiplied by 1 – Hct. Arterial Hct was substituted for IJV Hct in the plasma flow equation, which is further discussed in the “Methodological considerations” section of the manuscript. In the case of a measured negative fractional extraction of cerebral [^3^H]NA, we considered it as zero extraction on the ground of lack of plausibility.

### Statistical analysis

**Figure 2.**
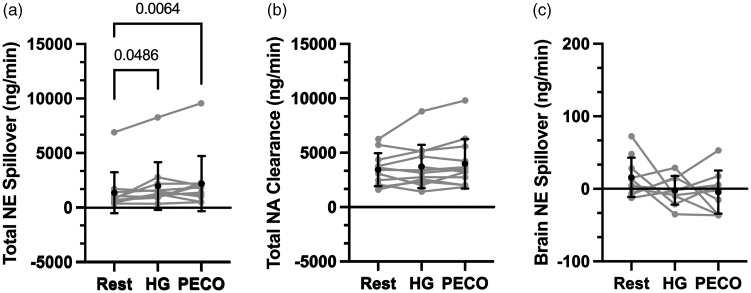
Isometric handgrip exercise on noradrenaline spillover and clearance. Mean ± SD and individual data for total noradrenaline spillover (Panel a; n = 11), total noradrenaline clearance (Panel b; n = 12), and brain noradrenaline spillover (Panel c; n = 10 [Rest], 9 [HG], 9 [PECO]). Statistical comparisons that yielded P < 0.05 are illustrated on the Figure.

**Figure 3.**
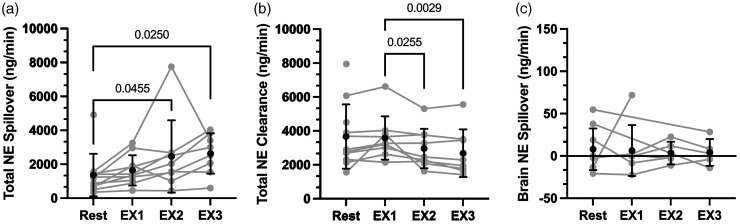
Supine cycling exercise on noradrenaline spillover and clearance. Mean ± SD and individual data for total noradrenaline spillover (Panel a; n = 11 [Rest], 10 [EX1], 9 [EX2], 8 [EX3]), total noradrenaline clearance (Panel b; n = 11 [Rest], 9 [EX1], 9 [EX2], 8 [EX3]), and brain noradrenaline spillover (Panel c; n = 9 [Rest], 7 [EX1], 5 [EX2], 5 [EX3]). Statistical comparisons that yielded P < 0.05 are illustrated on the Figure.

Statistical analyses were performed using GraphPad Prism V8.0 (San Diego, CA) and reported as means ±SD. Statistical significance was set at P < 0.05. Repeated measures one-way analysis of variance mixed effects models were used to detect statistical differences between rest, HG, and PECO (Tables 1, 2, and Figure 2), and rest, EX1, EX2, and EX3 (Tables 1, 2, and Figure 3). This statistical model accounts for the missing data, and when significant F-ratios were detected, post hoc comparisons were made using Tukey’s test. The effect size (i.e., Cohen’s d) values associated with our primary outcome (cerebral NA spillover) was computed using the estimated mean differences divided by the pooled standard deviation of the comparison.^20^

**Table 1.** Cardiorespiratory data during isometric handgrip exercise and post-exercise circulatory occlusion and supine cycling exercise.

	Rest	HG EX	PECO	Rest	Cyc EX1	Cyc EX2	Cyc EX3
IJV Diameter (mm)	9.1 ± 2.0	8.9 ± 2.1	8.6 ± 2.0	7.5 ± 1.8	7.2 ± 1.9	6.8 ± 1.8	8.6 ± 1.6
IJV Mean velocity (cm/s)	9.1 ± 3.4	12.4 ± 4.5*	11.7 ± 4.1	10.5 ± 3.3	10.0 ± 3.0	9.2 ± 1.9	7.8 ± 3.8
IJV Mean blood flow (ml/min)	338.5 ± 140.9	421.7 ± 108.8*	379.2 ± 127.3*	284.7 ± 153.1	276.1 ± 194.7	209.5 ± 114.0	255.2 ± 105.1
IJV Con (ml/min/mmHg)	3.4 ± 1.2	3.7 ± 1.0	3.4 ± 1.1	3.0 ± 1.5	2.7 ± 1.7	2.0 ± 1.1*	2.3 ± 0.8
Systolic BP (mmHg)	125.4 ± 12.1	140.9 ± 15.6*	135.6 ± 12.1*	123.9 ± 10.5	169.3 ± 15.1*	177.4 ± 20.4*	179.7 ± 18.2*
Diastolic BP (mmHg)	84.6 ± 8.2	97.4 ± 12.8*^‡^	92.2 ± 8.8*	78.2 ± 5.7	83.7 ± 9.1*	84.3 ± 7.7*	84.4 ± 5.8*
Mean BP (mmHg)	98.9 ± 9.8	117.0 ± 13.7*^‡^	110.0 ± 11.5*	93.5 ± 8.1	105.1 ± 12.0*	109.2 ± 11.3*	112.9 ± 11.5*^‡^
Heart rate (bpm)	66.6 ± 11.6	82.6 ± 11.3*^‡^	71.2 ± 11.4	62.5 ± 10.1	105.2 ± 8.4*	127.3 ± 13.4*^‡^	150.8 ± 16.2*^†^^‡^
Ventilation (l/min)	22.7 ± 4.5	33.8 ± 10.8*	27.1 ± 8.4				
Tidal volume (l)	1.2 ± 0.2	1.5 ± 0.5*	1.5 ± 0.2				
Breathing rate (breaths/min)	19.6 ± 1.9	22.7 ± 4.6^‡^	18.3 ± 4.4				
P_ET_CO_2_ (mmHg)	43.7 ± 2.0	44.3 ± 1.9	44.3 ± 1.8				
P_ET_O_2_ (mmHg)	86.6 ± 5.1	87.8 ± 4.2	88.2 ± 4.7				

BP: blood pressure; bpm: beats per minute; cm/s: centimeter per second; Con: conductance; HG EX: handgrip exercise; IJV: internal jugular vein; l: liters; l/min: liters per minute; ml/min: millimeter per minute; mm: millimeter; mmHg: millimeters of mercury; PECO: post-exercise circulatory occlusion; P_ET_CO_2_: Partial pressure of end-tidal carbon dioxide; P_ET_O_2_: Partial pressure of end-tidal oxygen. *P < 0.05 vs rest. ^†^P < 0.05 vs Cyc EX2. ^‡^P < 0.05: HG EX vs PECO, and vs Cyc EX1.

**Table 2.** Arterial and venous blood data during isometric handgrip exercise and post-exercise circulatory occlusion and supine cycling exercise.

	Rest	HG EX	PECO	Rest	Cyc EX1	Cyc EX2	Cyc EX3
IJV NA (pg/ml)	468.6 ± 441.0	576.0 ± 457.1	585.9 ± 466.8	467.2 ± 442.6	465.8 ± 323.1	997.3 ± 813.4	1275.3 ± 831.3*^‡^
ART NA (pg/ml)	404.5 ± 412.4	593.1 ± 409.1*	638.0 ± 475.3	419.9 ± 300.5	480.4 ± 309.1	913.6 ± 691.9	1259.0 ± 838.0*^‡^
A-V NA (pg/ml)	−64.1 ± 115.6	17.1 ± 90.7	52.1 ± 146.6	−47.3 ± 184.3	−13.4 ± 109.3	−25.0 ± 177.7	−91.7 ± 324.5
ART [Hb] (g/dl)	14.9 ± 1.2	14.8 ± 1.1	14.6 ± 1.3	14.7 ± 1.2	14.8 ± 1.3*	15.2 ± 1.4*^‡^	15.3 ± 1.2*^‡^
ART Hct (%)	45.6 ± 3.7	45.3 ± 3.5	44.9 ± 4.0	45.1 ± 3.6	45.5 ± 3.9*	46.5 ± 4.2*^‡^	46.9 ± 3.7*^‡^
CvO_2_ (ml/dl)	14.2 ± 1.8	14.6 ± 1.7	13.6 ± 2.5	13.1 ± 1.9	13.4 ± 1.5*	13.3 ± 1.5	12.3 ± 1.7^†^^‡^
CaO_2_ (ml/dl)	19.7 ± 1.6	19.7 ± 1.5	19.5 ± 1.7	19.6 ± 1.6	19.8 ± 1.7	20.2 ± 1.8*^‡^	20.4 ± 1.6*^‡^
A-V O_2_ content (ml/dl)	5.5 ± 1.3	5.1 ± 1.2	5.9 ± 1.6	6.5 ± 1.7	6.4 ± 0.9	6.9 ± 1.0	8.1 ± 1.1*^†^^‡^
IJV SO_2_ (%)	70.9 ± 4.2	72.7 ± 5.6	67.3 ± 9.8	64.6 ± 7.3	65.4 ± 3.7	62.6 ± 6.9	58.3 ± 5.9*^†^^‡^
ART SO_2_ (%)	97.3 ± 0.6	97.8 ± 0.6	97.6 ± 0.7	97.8 ± 0.4	97.8 ± 0.4	97.8 ± 0.7	98.0 ± 0.5
A-V SO_2_ (%)	26.3 ± 4.7	25.1 ± 5.8	30.3 ± 10.2	33.2 ± 7.2	32.4 ± 3.9	35.2 ± 7.3	39.7 ± 6.1*^†^^‡^
IJV PO_2_ (mmHg)	43.1 ± 4.3	44.3 ± 4.8	40.3 ± 7.0	38.2 ± 4.4	38.6 ± 2.8	37.4 ± 2.7	35.7 ± 2.8^‡^
ART PO_2_ (mmHg)	95.7 ± 5.2	102.1 ± 8.4	102.8 ± 13.7	102.1 ± 7.1	102.8 ± 8.1	100.1 ± 6.1	106.5 ± 7.7*^†^
A-V PO_2_ (mmHg)	52.6 ± 7.6	57.8 ± 10.7	62.6 ± 18.5	63.9 ± 5.9	64.2 ± 7.8	62.7 ± 6.3	70.8 ± 9.2*^†^^‡^
IJV PCO_2_ (mmHg)	52.1 ± 4.4	52.5 ± 4.0	51.1 ± 4.1	51.1 ± 3.1	50.8 ± 3.4	49.6 ± 4.5*	47.5 ± 5.0*^†^^‡^
ART PCO_2_ (mmHg)	44.6 ± 2.5	43.4 ± 3.3	42.0 ± 6.1	40.5 ± 2.5	40.2 ± 3.3	38.3 ± 4.2*^‡^	34.9 ± 3.8*^†^^‡^
A-V PCO_2_ (mmHg)	−7.5 ± 2.9	−9.1 ± 3.2	−9.1 ± 4.1	−9.7 ± 3.8	−10.6 ± 1.2	−11.2 ± 1.2	−12.6 ± 2.1*^‡^
IJV pH	7.34 ± 0.02	7.35 ± 0.02	7.35 ± 0.02	7.35 ± 0.01	7.34 ± 0.02	7.34 ± 0.02*^‡^	7.33 ± 0.02*^‡^
ART pH	7.38 ± 0.02	7.39 ± 0.02	7.39 ± 0.05	7.40 ± 0.02	7.39 ± 0.02	7.39 ± 0.02	7.39 ± 0.02
A-V pH	0.04 ± 0.01	0.05 ± 0.01	0.04 ± 0.03	0.05 ± 0.01	0.05 ± 0.01	0.05 ± 0.01	0.06 ± 0.01
IJV Glucose (mmol/l)	5.4 ± 0.8	5.6 ± 1.0	5.5 ± 1.1	5.1 ± 0.4	5.1 ± 0.2	5.1 ± 0.3	5.0 ± 0.6
ART Glucose (mmol/l)	6.0 ± 1.2	6.0 ± 1.0	6.1 ± 1.0	5.6 ± 0.3	5.6 ± 0.3	5.6 ± 0.3	5.7 ± 0.6
A-V Glucose (mmol/l)	0.6 ± 0.6	0.4 ± 0.1	0.6 ± 0.2	0.5 ± 0.2	0.6 ± 0.2	0.5 ± 0.1	0.7 ± 0.2*^†^^‡^
IJV Lactate (mmol/l)	0.7 ± 0.1	0.8 ± 0.2	0.8 ± 0.4	1.0 ± 0.6	1.9 ± 0.9	3.3 ± 1.9*^‡^	4.8 ± 1.8*^†^^‡^
ART Lactate (mmol/l)	0.6 ± 0.1	0.8 ± 0.2	0.8 ± 0.5	0.9 ± 0.7	1.9 ± 1.0	3.4 ± 2.1*^‡^	5.2 ± 2.2*^†^^‡^
A-V Lactate (mmol/l)	−0.04 ± 0.09	−0.02 ± 0.08	0.02 ± 0.10	−0.08 ± 0.15	−0.00 ± 0.03	0.1 ± 0.4	0.4 ± 0.7*

ART: arterial; CaO_2_: arterial oxygen content; CvO_2_: venous oxygen content; g/dl: grams per deciliter; [Hb]: hemoglobin concentration; Hct: hematocrit; HG EX: handgrip exercise; IJV: internal jugular vein; ml/dl: milliliters per deciliter; mmHg: millimeters of mercury; mmol/l: millimole per liter; NA: noradrenaline; pg/ml: picogram per millimeter; PCO_2_: partial pressure of carbon dioxide; PECO: post-exercise circulatory occlusion; PO_2_: partial pressure of oxygen; SO_2_: oxygen saturation. *P < 0.05 vs rest. ^†^P < 0.05 vs Cyc EX2. ^‡^P < 0.05: HG EX vs PECO, and vs Cyc EX1.

## Results

### Participants

The participants included in this study data analysis (n = 12; 5 females) had a mean ± SD age of 25.7 ± 2.8 years, height of 172.2 ± 8.4 cm, and body mass of 71.7 ± 13.8 kg. Our primary outcome measure, cerebral NA spillover, was obtained in only a portion of participants during the HG + PECO protocol (n = 9), and cycling exercise EX1 (n = 7), EX2 (n = 5), and EX3 (n = 5). Participant dropout was primarily due to inadequate image quality during exercise. Individual data for NA spillover and clearance are illustrated in Figures 2 and 3.

### The effects of exercise on cardiovascular and respiratory variables

Table 1 illustrates cardiovascular and respiratory data during exercise. During isometric HG and PECO, IJV diameter remained unchanged (P = 0.748), but IJV blood velocity was elevated during HG (P = 0.0229); and IJV blood flow was elevated during both isometric HG (P = 0.0035) and PECO (P = 0.0359) compared to rest. During isometric HG and PECO, systolic (P < 0.01), diastolic (P < 0.001), and mean arterial blood pressure (P < 0.001) all increased compared to rest; however, IJV conductance was unchanged (P = 0.251). Heart rate was only elevated during isometric HG (P < 0.001), but not during PECO (P = 0.0658). Minute ventilation and tidal volume were elevated during isometric HG (P < 0.001 and P = 0.0318, respectively), but not during PECO (P = 0.13 and P = 0.06, respectively). Breathing frequency did not change between rest and isometric HG (P = 0.09) or PECO (P = 0.59), but it was elevated during isometric HG compared to PECO (P = 0.0146). Both P_ET_CO_2_ and P_ET_O_2_ were controlled to resting values during isometric HG and PECO (P = 0.122 and P = 0.136, respectively).

During supine cycling exercise, IJV diameter, blood velocity, and blood flow did not change compared to rest (P = 0.291, P = 0.126, and P = 0.141, respectively). Systolic, diastolic, and mean arterial blood pressure were all elevated compared to rest (all P < 0.001), and IJV conductance was reduced during EX2 compared to rest (P = 0.0459). Heart rate was elevated at each exercise intensity compared to rest (P < 0.001).

### The effects of exercise on arterial and venous blood

Table 2 illustrates arterial and venous blood data. Arterial NA was elevated during isometric HG (P = 0.0237), but not PECO (P = 0.0848), and there were no changes in IJV NA during isometric HG and PECO (main effect: P = 0.547) compared to rest. During isometric HG and PECO, there were no differences found for arterial or venous [Hb], oxygen content, oxygen saturation, PO_2_, PCO_2_, pH, glucose, lactate and arterial Hct (all P > 0.05).

Arterial NA and IJV NA were elevated at EX3 compared to both rest and EX1 (all P < 0.01). Both arterial [Hb] and Hct were elevated during supine cycling exercise compared to rest (main effects: P < 0.04). Internal jugular C_V_O_2_, SO_2_, PO_2_, and PCO_2_ all declined with supine cycling exercise (main effects: P < 0.05), while arterial CaO_2_, and PO_2_ increased compared to rest (P < 0.05). Arterial SO_2_ remained unchanged (main effect: P = 0.643), and arterial PCO_2_ decreased (P < 0.001). Internal jugular pH decreased during supine cycling exercise (main effect: P < 0.001), but arterial pH remained the same (main effect: P = 0.05). Internal jugular and arterial glucose did not change with supine cycling exercise (main effects: P = 0.907 and P = 0.818, respectively), but IJV and arterial lactate increased (main effects: P < 0.001).

### The effects of exercise on noradrenaline spillover and clearance

Figure 2 illustrates NA data during isometric HG exercise. Total NA spillover was elevated during isometric HG (P = 0.049) and persisted during the PECO (P = 0.006); however, NA clearance remained unchanged compared to rest (P = 0.130). Cerebral NA spillover remained unchanged during isometric HG exercise (P = 0.36; Cohen’s d = 0.46) and during the PECO (P = 0.45; Cohen’s d = 0.40), compared to rest.

Figure 3 illustrates NA data during supine cycling exercise. Total NA spillover during supine cycling exercise was higher during EX2 and EX3 compared to rest (P = 0.046, and P = 0.025, respectively). NA clearance was reduced at EX2 and EX3 compared to EX1 (P = 0.026 and P = 0.003, respectively). Despite changes in total NA spillover and clearance, cerebral NA spillover remained unchanged during supine cycling exercise (main effect: P = 0.94; Cohen’s d = 0.10).

## Discussion

Our study yielded several key findings: 1) in support to our initial hypothesis, cerebral NA spillover remained unchanged during isometric HG exercise and PECO, and supine cycling exercise; and 2) total NA spillover increased during isometric exercise and PECO, as well as during dynamic supine cycling exercise, compared to rest. Taken together, these results indicate that transient increases in arterial blood pressure induced by acute moderate exercise does not engage cerebral SNA in healthy humans. We discuss the implications of these results in detail in the sections below.

### Sympathetic nerve activity during transient increases in arterial blood pressure

It is well-established that both isometric and dynamic exercise lead to an increase in heart rate, arterial blood pressure, and peripheral SNA. Traditionally, SNA signaling to the peripheral vasculature results in increased smooth muscle tone (i.e., vasoconstriction). The mechanisms behind this involve a complex interplay between central command,^21^ afferent signaling from mechanical and metabolic sensory receptors in skeletal muscles,^22^ feedback from stretch receptors in the carotid arteries and aortic arch,^23^ low pressure stretch receptors in the heart, great veins, and pulmonary vessels,^24^ arterial chemoreceptors,^25^ and phrenic activity.^26^ Microneurography and plasma NA are commonly used experimental techniques to measure SNA during both isometric (e.g., HG) and dynamic (e.g., cycling) exercise. Previous research has demonstrated that peripheral SNA robustly increases and even persists during PECO.^27–31^ Our study's findings are consistent with the current body of literature, as we observed an increase in total NA spillover during both isometric (Figure 2) and dynamic exercise (Figure 3).

The question of whether peripheral SNA accurately reflects cerebral SNA in humans at rest, or during physiological stress (e.g., exercise) has been a topic of controversy. This is because different vascular beds in the human body have varying distributions of sympathetic neurotransmitter receptor densities,^32^ and possibly different sensitivities. Thus, it is plausible – and perhaps even likely – that the cerebrovasculature responds differently compared to vascular beds located in the periphery given its unique features and functions, such as the circle of Willis and the brain confinement to the human skull. From a teleological standpoint, it would be physiologically beneficial for cerebral SNA to increase similarly to peripheral SNA in response to transiently elevated arterial blood pressure during exercise as a protective mechanism against overperfusion. Indeed, this hypothesis is supported by animal models that have demonstrated increases in SNA during acute increases in arterial blood pressure.^5,33^ However, there is evidence of interspecies differences in CBF regulation,^33–35^ which underscores the importance of conducting cerebral NA spillover studies in humans. The limited data collected in healthy humans suggests that peripheral and cerebral SNA are similar, at least under resting conditions.^36^ The data from the current study highlights that in contrast to available animal data, and in support of the limited data collected in healthy humans,^7^ transient increases in arterial blood pressure during moderate intensity isometric and dynamic exercise had no impact on cerebral SNA.

### The influence of sympathetic nerve activity on the cerebrovasculature

The extent to which SNA regulates the cerebrovasculature remains under scrutiny,^37,38^ despite the rich innervation of the cerebral circulation by both sympathetic and parasympathetic nerve fibers. Various pharmacological blockades and ganglion excision studies conducted in humans over the past few decades have shown that altering sympathetic signaling to the cerebrovasculature can affect resting CBF and reactivity to physiological stressors such as carbon dioxide and transient decreases and increases in arterial blood pressure.^37,38^ However, the interpretation of these studies is challenging due to methodological limitations associated with the use of transcranial Doppler ultrasound and the simultaneous effects of drug interactions on physiological parameters that can directly influence cerebrovascular tone, such as arterial blood pressure and cardiac output.

#### Cerebral sympathetic nerve activity during moderate intensity isometric handgrip exercise and post-circulatory occlusion

During aerobic exercise, cardiovascular adjustments are necessary to ensure sufficient oxygen and nutrient supply to active skeletal muscle tissue and active brain regions, resulting in a 10–20% increase in cerebral perfusion.^39^ However, it remains unclear whether SNA-mediated protective mechanisms for overperfusion are present at these modest levels of transient increases in arterial blood pressure during isometric and dynamic exercise. Surprisingly, brain NA spillover remained unchanged during isometric exercise, indicating that SNA is not a regulatory mechanism for the cerebrovasculature during isometric exercise as used in the current study. Only one study has investigated the sympathetic control of CBF in healthy humans using pharmacological intervention (i.e., prazosin) during isometric HG exercise,^10^ which found that regional sympathetic neural restraint may be present. The discrepancy between our findings and previous research may be due to the NA spillover technique providing a whole brain measurement of SNA, rather than measuring a regional response. One interesting finding was that brain NA spillover also remained unchanged during PECO, after the removal of central command (i.e., voluntary muscle contraction). Future work should focus on the effects of non-exercise induced sympathetic stress on cerebral NA spillover.

#### Cerebral sympathetic nerve activity during mild-to-moderate intensity supine cycling exercise

Similar to previous work,^7^ we found that brain NA spillover remained unchanged during mild-to-moderate intensity supine cycling exercise. Other studies have investigated the effects of SNA on CBF regulation during dynamic exercise using pharmacological interventions. Two studies used β-adrenergic blockades (metoprolol and propranolol) and found that they had no effect on CBF regulation at rest or during cycling exercise.^40,41^ Another study measured the impact of ganglion blockade, which has demonstrated to reduce CBF at rest,^42^ during cycling exercise with simultaneous administration of a β-adrenergic blockade (e.g., metoprolol) or placebo. The data from the latter study indicate that under cycling exercise conditions where elevations in cardiac output are limited (e.g., after β-adrenergic blockade), global SNA signalling increases ∼2–3x (measured via arterial NA), and is associated with a reduction in the CBF response to exercise.^43^ The complex pharmacological interventions and extreme conditions of limited cardiac output during dynamic exercise make these studies difficult to compare to the current study, but taken together, submaximal dynamic exercise in itself may not be a strong enough physiological stressor to elicit profound increases in cerebral SNA.

### Methodological considerations

Although these are the only available data on cerebral NA spillover during both isometric and dynamic exercise, there are several methodological limitations that require further discussion. First, this invasive study includes a small sample size of healthy males and females and some data were lost during the protocol, primarily due to inadequate image quality during exercise. This work cannot be generalized to older participants or clinical populations (e.g. patients with arterial hypertension). Second, the current findings do not exclude the notion cerebral SNA acts as a protective mechanism for the cerebrovasculature in response to increases in arterial blood pressure in other physiological/clinical conditions. Indeed, it was not possible to measure cerebral SNA during transient surges in arterial blood pressure, but only following relatively slow steady-state increases (i.e. over 2 (HG exercise) to 3 (supine cycling exercise) minutes). A strength to the study is that HG and cycling exercise yielded similar increases in mean arterial blood pressure (18.3 and 20.7%, respectively). However, the duration and nature of the stimulation may influence the cerebrovascular response and SNA control of the cerebral circulation may be more efficient under dynamic than steady-state conditions.^37,44^ Third, the participants underwent various stressful procedures and interventions, including catheter placements, exercise, multiple ultrasound methods, and had to breathe on a custom end-tidal forcing system, resulting in resting arterial and venous NA concentrations that were higher than anticipated. However, the individual trends in NA concentrations (and calculated whole body NA spillover) in response to exercise were consistent with previous research. Fourth, IJV Hct, a parameter included in the calculation of IJV plasma flow, was not included in our panel of blood parameters analyzed, although arterial Hct was. We have substituted IJV Hct for arterial Hct in the plasma flow equation. Considering venous Hct is ∼3% higher than arterial Hct,^45^ our cerebral NA spillover values will be slightly overestimated. However, this should not have impacted within-participant comparisons. Fifth, we attempted to take measurements of CBF (e.g., internal carotid artery blood flow) during the exercise protocols, but due to the difficulty of obtaining simultaneous CBF and IJV blood flow, we decided to solely focus on the IJV measurements for the calculation of cerebral NA spillover. Future studies using cerebral NA spillover should aim to collect these complementary data to gain a full scope of the cerebrovascular response to physiological stressors. Sixth, due to the nature of the experimental measurements taken and the possibility of IJV collapse in the upright position, submaximal exercise was completed in the supine position, which could impact the performance and the physiological response to cycling exercise. Seventh, there is evidence that regional NA spillover underestimates true NA release, therefore, small changes in SNA via NA spillover may be difficult to detect.^46^ Eighth, testing participants in the supine position loads cardiopulmonary baroreceptors, which may influence the sympathetic nerve response to exercise. Lastly, the cerebrovasculature is richly innervated by parasympathetic nerve fibers, yet our data do not account for any contribution of parasympathetic nerve activity on the cerebrovascular function. This is a crucial area of research that should be addressed in future studies.

### Perspectives

These results represent a key milestone towards the examination of the impact of exercise on cerebral SNA in healthy humans. Further research aimed at improving our understanding of the role of SNA on cerebrovascular regulation in several clinical conditions associated with central sympathetic overactivity such as ischemic heart disease, chronic heart failure, autonomic disorders, and arterial hypertension are needed. Furthermore, the exercise stressors conducted in this study yield moderate elevations of peripheral SNA, and more powerful sympathetic stimuli should be explored in future studies in healthy humans. A better understanding of the physiological role of SNA on the cerebrovasculature during different physiological and clinical conditions in humans may also provide the basis for: 1) developing new approaches to pharmacologically manage pathological conditions characterized by cerebral sympathetic dysregulation and impaired CBF control and, 2) new therapeutic targets for early detection and prevention of cerebral hemorrhage/ischemic injuries and better management of CBF during anesthesia or in intensive care.

## Conclusion

This is the first investigation to measure brain specific sympathetic nerve activity using the noradrenaline spillover technique during isometric and dynamic exercise in humans. The primary finding was that cerebral noradrenaline spillover remained unchanged during isometric (i.e., handgrip), post exercise cuff occlusion, and dynamic (i.e., cycling) exercises. The data from the current study opposes the available animal literature but are largely in support of the available healthy human data. Future research should focus on the potential effects of other mild-to-strong sympathetic stressors on cerebral sympathetic nerve activity in both healthy and disease populations to gain a better understanding of its role within the cerebrovasculature.
